# “I know it’s bad for me and yet I do it”: exploring the factors that perpetuate smoking in Aboriginal Health Workers - a qualitative study

**DOI:** 10.1186/1472-6963-12-102

**Published:** 2012-07-11

**Authors:** Anna P Dawson, Margaret Cargo, Harold Stewart, Alwin Chong, Mark Daniel

**Affiliations:** 1 Social Epidemiology and Evaluation Research Group, Sansom Institute for Health Research, University of South Australia, Adelaide, South Australia, Australia; 2 Aboriginal Health Council of South Australia, Adelaide, South Australia, Australia

## Abstract

**Background:**

Aboriginal Health Workers (AHWs) have a mandate to deliver smoking cessation support to Aboriginal people. However, a high proportion of AHWs are smokers and this undermines their delivery of smoking cessation programs. Smoking tobacco is the leading contributor to the burden of disease in Aboriginal Australians and must be prevented. Little is known about how to enable AHWs to quit smoking. An understanding of the factors that perpetuate smoking in AHWs is needed to inform the development of culturally relevant programs that enable AHWs to quit smoking. A reduction of smoking in AHWs is important to promote their health and also optimise the delivery of smoking cessation support to Aboriginal clients.

**Methods:**

We conducted a fundamental qualitative description study that was nested within a larger mixed method participatory research project. The individual and contextual factors that directly or indirectly promote (i.e. perpetuate) smoking behaviours in AHWs were explored in 34 interviews and 3 focus groups. AHWs, other health service staff and tobacco control personnel shared their perspectives. Data analysis was performed using a qualitative content analysis approach with collective member checking by AHW representatives.

**Results:**

AHWs were highly stressed, burdened by their responsibilities, felt powerless and undervalued, and used smoking to cope with and support a sense of social connectedness in their lives. Factors directly and indirectly associated with smoking were reported at six levels of behavioural influence: personal factors (e.g. stress, nicotine addiction), family (e.g. breakdown of family dynamics, grief and loss), interpersonal processes (e.g. socialisation and connection, domestic disputes), the health service (e.g. job insecurity and financial insecurity, demanding work), the community (e.g. racism, social disadvantage) and policy (e.g. short term and insecure funding).

**Conclusions:**

An extensive array of factors perpetuated smoking in AHWs. The multitude of personal, social and environmental stressors faced by AHWs and the accepted use of communal smoking to facilitate socialisation and connection were primary drivers of smoking in AHWs in addition to nicotine dependence. Culturally sensitive multidimensional smoking cessation programs that address these factors and can be tailored to local needs are indicated.

## Background

Nicotine ingestion has a long history in Aboriginal communities. In traditional (pre-colonial) society it was common practice for Aboriginal people to chew wild plants containing nicotine, known as *pituri*. The intoxicating effects of pituri lead to its prominence in tribal life in social interactions and as a bartering commodity [1]. Social control mechanisms limiting consumption of nicotine were employed, however, by constraining the production and distribution of nicotine-bearing plants. These mechanisms were lost with the advent of colonisation whereupon widespread and frequent exposure to introduced tobacco occurred through rations and wage payments. Aboriginal people rapidly became addicted to the physiological effects of tobacco, and this addiction was exploited by colonists who desired cheap labour and local knowledge [2].

Smoking tobacco remains common among contemporary Aboriginal peoples. In 2008, close to half (47%) of Aboriginal and Torres Strait Islanders ≥15 years of age were current daily smokers [3]. Though this prevalence represents a slight though statistically significant decline in comparison to previous surveys of Aboriginal peoples [4], it remains more than double that of the non-Aboriginal population [3]. Tobacco is the leading contributor to the burden of disease in Aboriginal Australians (equating to 12% of the total burden) [5] and as such is described by the Australian government as “the single biggest killer of Indigenous people”(p.6) in Australia [6]. A reduction in smoking prevalence has great potential to improve the health and wellbeing of Aboriginal peoples. The Australian government’s goal of ‘Closing the Gap’ in life expectancy between Indigenous and non-Indigenous Australians within a generation has led to a significant financial investment to tackle Indigenous smoking [6]. A culturally relevant evidence base is needed, however, to inform the development and delivery of appropriate smoking cessation and support services.

Aboriginal Health Workers (AHWs) have a mandate to deliver holistic health care to Aboriginal communities and are often the first person an Aboriginal person sees when accessing primary health care services [7]. AHWs have Certificate IV training in primary health care, or equivalent practical experience, and are employed in both community-controlled Aboriginal health services (governed by a board of local Aboriginal community members) and government-controlled health services (governed by the state health department). AHWs have diverse responsibilities within the health service including, for example, providing vaccinations and health checks, delivering outreach services to the wider community, and coordinating a variety of health programs including smoking cessation programs [8]. AHWs have a pivotal role in breaking the cycle of smoking in Aboriginal communities. In addition to promoting smoking cessation, they are viewed as role models by their peers [9]. These two primary responsibilities, however, are undermined by the high prevalence of smoking in AHWs. Studies with AHWs in the last decade report alarming smoking prevalence rates in the range of 46-64% across Australia [10-14].

The smoking status of AHWs has been found to influence the services they provide to the community. A recent review of barriers to the delivery of tobacco interventions by AHWs suggests being a smoker is a key obstacle [15]. AHWs report that smoking cigarettes makes them less likely to provide smoking cessation advice to Aboriginal clients [11,12,16,17], and attribute this to a fear of feeling hypocritical [11]. Since the smoking behaviours of AHWs inhibits the smoking cessation advice they provide to Aboriginal clients, prevention or reduction of smoking in AHWs could potentially lead to improved tobacco related service provision and, in turn, reduced smoking rates in the Aboriginal population. This population benefit is in addition to the resultant health benefits for AHWs themselves.

Little is known about how to enable AHWs to quit smoking. Mark and co-authors [11] conducted a single focus group discussion with AHWs and found that stress, gambling and drinking were associated with smoking. To date, no published studies comprehensively examine the factors that influence smoking in AHWs. An understanding of the factors that directly or indirectly promote (i.e. perpetuate) smoking behaviours in AHWs is needed to inform the development of culturally relevant programs that enable AHWs to quit smoking. The aim of this study, therefore, was to explore the perceptions of AHWs, their managers and co-workers in relation to the individual and contextual factors that are associated with smoking in AHWs.

## Methods

### Study context

This qualitative study was underpinned by the six Iga Warta principles that ensure Aboriginal research projects take a pro-active preventive approach, coordinate activities across sectors, address issues of sustainability, consider the social determinants of health, and are sensitive to Aboriginal notions of time and space and Aboriginal community and family. To uphold these principles, the project took a participatory research approach with university researchers and key members of the Aboriginal Health Council of SA (AHCSA) sharing decision making throughout the project. Ongoing support was provided by the State-wide Puyu Wiya (No Smoking) Advisory Group and the Aboriginal Primary Health Care Workers Forum. These two groups are peak bodies for decision-making related to tobacco prevention and control and for supporting Aboriginal Health Workers, respectively. The presence of mutual trust and respect among all partners was important to support two-way learning, empowerment, shared ownership and to ensure that culture was respected in the research process [18].

### Study design and theoretical framework

A fundamental qualitative descriptive design [19] was utilised to enable a comprehensive, low-inference description of phenomena for the purpose of informing program development. The study was guided by social ecological theory [20] which provides a set of principles for understanding the “interrelations among diverse personal and environmental factors in human health and illness” (p. 283) [21]. A social ecological perspective was selected since it is consistent with Aboriginal notions of health and wellbeing [22]. The social ecological framework recognises that behaviour is affected by multiple levels of influence including personal factors, interpersonal processes, organisational factors, community factors and public policy [23]. Given the central role of the family in traditional and contemporary Aboriginal living [24], the framework was adapted to include family as a unique and proximal level of influence. In the context of AHWs, the ‘organisation’ level was the health service in which AHWs were employed. Hence, the factors associated with smoking in AHWs were framed in six levels of behavioural influence: personal factors, family, interpersonal processes, the health service, the community and public policy.

### Data collection and study sample

We pursued a purposive sampling plan that included AHWs with a range of smoking histories (never smoked, current smoker, ex-smoker) in addition to health service managers and tobacco control personnel. A balanced gender representation was considered desirable, as was a varied geographical distribution of respondents (including staff from metropolitan, regional and remote health services). The views of non-AHWs (i.e. health service management, tobacco control personnel and other health staff) were sampled to gain further insight into the broader socio-ecological (e.g. interpersonal, organisational, community and policy level) features that could influence AHWs’ smoking behaviours. In applying this sampling framework, we captured the direct experience of smoking from AHWs and utilised shadowed data’ in two ways to enhance sampling analysis [25]. During interviews AHWs referred to other AHWs sharing similar experiences (‘generalised self’) and those not like them (‘generalised other’). In addition, perceptions of those external to AHWs (i.e., health service and tobacco control personnel) were included to “make the domain and various components of the phenomenon understandable more quickly” [25] (pg. 291). Here, external perspectives provided insight into the range of social-ecological factors perpetuating AHW smoking.

Since relationships and trust are central to working respectfully with Aboriginal communities, the researchers sought the mentorship of the State-wide Tackling Tobacco Coordinator who was a respected elder employed at AHCSA. He championed the project in his role and acted as a cultural mentor for the researchers. Health services that employed AHWs were contacted including both community-controlled Aboriginal health services and government-controlled health services. Fifteen site visits to metropolitan, regional and remote health services were undertaken in conjunction with the Tackling Tobacco Coordinator where possible, to provide the research team and the project with credibility. The recruitment strategy was guided by two Aboriginal members of the investigating team who advised when to visit health services based upon their knowledge of local challenges, opportunities and competing organisational demands. During site visits, the team presented information about the research project and engaged in informal discussions about smoking with local health staff. As a sense of trust developed, AHWs and health service management were invited to formally participate in the study. Tobacco control personnel were recruited individually (via telephone) and interviewed in the workplace in one instance and via telephone in another instance. Recruitment continued until the sampling plan had been achieved and data saturation occurred.

Semi-structured interviews were the primary data source. Thirty-four interviews were conducted between August 2009 and August 2010, including 20 interviews undertaken with government-controlled health service employees and 14 interviews undertaken with community-controlled health service employees. Interviews were conducted until no new data emerged, with the final three interviews yielding no new information. Interview duration (mean 51 [range 16–133] minutes) was dependent upon availability of time and readiness to share information. Table 1 describes the 23 AHWs, 9 other health service staff and two tobacco control personnel that participated.

**Table 1:** Participant characteristics of stakeholders who participated in interviews (n = 34)

	Aboriginal Health Workers	Health Service Personnel (Managers, Supervisors, Nursing staff)	Tobacco Control Personnel	All
Participants	23	9	2	34
Gender (M/F)	10/13	3/6	2/0	15/19
Location				
Metropolitan	9	2	1	12
Rural	13	6	1	20
Remote	1	1	0	2
Ethnicity				
Aboriginal	23	3	2	28
Other	0	6	0	6
Smoking History				
Never Smoked	1	5	0	6
Ex-smoker				
Action	3	0	0	3
Maintenance	6	0	2	8
Current smoker				
Precontemplation	3	0	0	3
Contemplation	7	3	0	10
Preparation	3	1	0	4

There were five interview schedules developed to guide semi-structured interviews, including four for AHWs with a smoking history (i.e. ex-smokers, smokers not ready to quit, smokers unsure about quitting, and smokers ready to quit) and a final one for both non-AHWs and AHWs who had never smoked. The interview schedules enquired into AHWs’ personal history of smoking (e.g. *Can you tell me how you started smoking?*), their reasons for smoking (e.g. *Can you tell me about why you smoke now?*) in addition to the situational circumstances of smoking for typical week and weekend days including when they smoked, with whom, where the smoking occurred, and the factors that triggered smoking (e.g. *Can you walk me through a typical day during the week to describe your smoking, starting from when you get up?*). Questions further explored experiences with quit attempts. Quitters were asked to reflect on their previous smoking history and discuss their experiences of quitting. Health service managers and AHWs who had never smoked were asked about smoking in the workplace, using questions such as “*Do many people at the health centre smoke? Where and when do they smoke?*”. The interviewers used clarificatory, exploratory, amplificatory and explanatory probes [26] to understand the smoking experience and the meaning ascribed to the individual, social, cultural and environmental factors related to smoking.

Focus group discussions were used to clarify emergent findings from the analysis of interview data and provide greater insight into understanding the organisational, social and community contexts related to smoking. The dynamic of group discussions provided a collective perspective regarding smoking in AHWs and the acceptability of smoking in the health service that was not illuminated in individual interviews. The discussion schedule for the focus groups was compiled after preliminary analysis of the first 22 interviews and was internally reviewed by an Aboriginal team member. Participation by AHWs as well as a range of other health service staff was welcomed in order to capture a broad range of perspectives. To recruit participants to focus groups, we visited health services, presented information about the study, and then invited all staff present to participate in a group discussion around smoking. In total, 17 health service staff (including 4 AHWs, 3 home and community care workers, 2 trainee enrolled nurses and 8 other health service staff) who were 53% female participated in three focus groups (mean 57 [range 45–71] minutes) conducted in regional locations in April and May 2010.

The interviewers were non-Aboriginal non-smokers who were experienced in conducting individual and focus group interviews and in undertaking research in Aboriginal communities. Interviews and focus groups were digitally recorded and transcribed verbatim by an external provider. Audio recording was not mandatory, and two participants elected not to be recorded. Further, one interview with a regional participant was conducted over the telephone. In these instances notes taken by the interviewer were used in analysis. All volunteers were provided with information about the nature of the research and gave written informed consent. Ethical approval for the study was granted from the Aboriginal Health Research and Ethics Committee, Aboriginal Health Council of South Australia, in addition to the Human Research Ethics Committees of the University of South Australia and SA Health, Government of South Australia.

### Data analysis

Analysis was undertaken using Nvivo 8 software (QSR International Pty Ltd, 2008). First, digital recordings were reviewed to ensure that the mood and emotions of the respondents were held within the data during analysis. Next, a brief narrative summary was generated for each interviewee. Transcribed text from interviews and focus groups and notes taken during unrecorded interviews were then analysed using a qualitative content analysis approach, based on the method of Graneheim and Lundman [27]. Sections of transcribed text that dealt with similar issues or fields of inquiry were first divided into content areas. Next, data were divided into meaning units that included words, sentences or paragraphs related in content and context. Thereafter, categories were created that represented internally homogeneous and externally heterogeneous [28] groups of meaning units that expressed the manifest content of the data [27]. Following the advice of Kitzinger [29], the views of individuals were distinguished from group consensus. Once the manifest content of the data had been analysed, the underlying themes evident in and emerging from the data were also identified. Themes are “a thread of an underlying meaning through condensed meaning units, codes or categories, on an interpretative level” (p.107) [27].

The first author conducted an analysis of the full dataset. Next, MC undertook a secondary review of the subdivision of data and the content and labelling of each category. A series of discussions were held between AD and MC to refine the content, labelling and meaning of categories. Given that AD and MC are non-Aboriginal researchers, further review by Aboriginal team members and AHWs was paramount. Hence, AD and MC consulted with an Aboriginal investigator (AC) and the Tackling Tobacco Coordinator (HS) to review and refine the categories. AHW representatives who were members of the Aboriginal Primary Health Care Workers Forum were then presented with the findings and invited to make amendments. An example of a revision that occurred during this process was an amendment to a family-level factor from ‘*dis-integration of family’* to ‘*breakdown of family dynamics’*. Finally, collective clarification was sought regarding policy level factors at a meeting of the project’s Working Group (that included AHWs, health service chief executive officers, tobacco control personnel and other key personnel from tobacco-related community organisations). The active engagement of Aboriginal partners in the data interpretation process enriched the findings and acted to further strengthen relationships.

## Results

**Figure 1:**
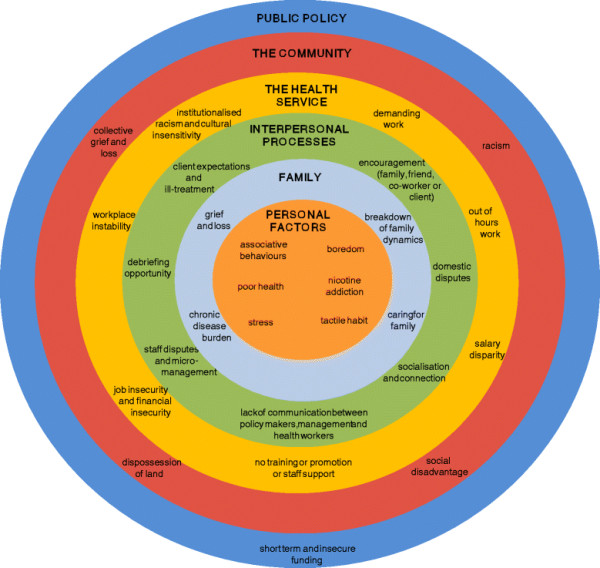
**A social ecological framework depicting the factors that directly or indirectly perpetuate smoking in Aboriginal Health Workers**. Factors associated with smoking in Aboriginal Health Workers were reported at six levels of behavioural influence in the adapted social ecological framework: personal factors, family, interpersonal processes, the health service, the community and public policy. The distribution of factors related to smoking in individual Aboriginal Health Workers was dependent upon their personal characteristics, relationships and environmental context

The stories shared during the interviews and focus groups highlighted an array of factors related to smoking in AHWs. Some factors were frequently or consistently identified, and others were unique to particular interviewees. Factors associated with smoking were depicted as (1) perceived environmental and individual stressors that elicit a stress response (i.e., frustration, anger, anxiety) in AHWs, (2) social norms or expectations that elicit socially acceptable behaviours in AHWs, (3) environmental cues that elicit a conditioned response in AHWs (e.g. drinking, gambling); and (4) a biological response to the addictive properties of nicotine. The former three classes of factors associated with smoking are indirect with their origins located in the environment while the latter class reflects a direct biological response that comes with the habituation of smoking. All these factors, whether direct or indirect, play a role in the maintenance of smoking, including smoking relapse. Figure 1 depicts the factors associated with smoking in AHWs, present at various levels of influence in the social ecological framework. In particular, it illustrates the range of factors related to smoking that exist beyond the level of the individual.

Given that the analysis sought to identify the factors that perpetuate smoking, and these were often related to life stressors, the findings presented here paint a somewhat pessimistic view of some aspects of Aboriginal society and experiences. As such, the discussions that follow neglect elaboration of the positive characteristics of Aboriginal life. This is not meant to imply a resounding negativity in relation to AHWs and their lives but rather reflects the biases and focus of the research. Indirect and direct factors associated with smoking are highlighted in ***bold italics*** in the text that follows and are contextualised with evidence from other population samples where relevant.

### Personal factors

***Stress*** emerged as a pervasive trigger for smoking in the stories shared by the participants. Sources of stress varied greatly; they included, for example, stress due to relationships and family issues, financial problems, community issues, and work challenges. Mark and colleagues [11] also identified stress was related to smoking in their small study of AHWs, and Lindorff et al. [30] found that stress was the primary reason why the general Aboriginal and Torres Strait Islander population smoke. AHWs spoke about smoking as a means to cope with stress:

"It just seems to relax me when I’m experiencing those high level stress situations, I know it’s bad for me and yet I do it."

Features of life present at the individual and five peripheral levels of influence (family, the health service, and so on) indirectly perpetuated smoking behaviours through increasing stress in AHWs. In these instances, smoking was used as a stress management strategy. For example, a small number of AHWs identified that they smoked as a means to cope with having ***Poor Health*** (e.g. anxiety, depression, chronic disease, back pain). As an AHW smoker described:

"It helps me deal with the pain stuff because the pain makes me feel more agro and niggly towards myself and I get a bit weird and just snapping at other people and in my head I have convinced myself that a cigarette will stop me from doing that and calm me down."

Several ***Associative Behaviours*** triggered AHWs to smoke such as getting in a car, watching television, and drinking alcohol or caffeinated drinks (i.e. coffee, coke). Even going outside prompted the desire to smoke in some AHWs. Furthermore, a ***Tactile Habit*** was mentioned by some AHWs as a reason for smoking.

"As soon as I get home and I put a coffee on or have wine or something, a beer or whatever, I want a smoke. So it must be something associated just with drinking or something in my hand."

Participants frequently identified ***Boredom*** as a trigger for smoking. Given that AHWs also spoke about high work demands, the somewhat contradictory notion of boredom as a reason for smoking was further explored in focus group discussions. It became apparent that “boredom” was used to represent having time on ones hands, such as when waiting for a client. It is possible that awareness of nicotine cravings heighten in idle times, which triggers the desire to smoke. Relief of boredom is a key smoking motive in the general population, and is positively correlated with both daily cigarette consumption and nicotine dependence [31].

Patterns of tobacco use were highly variable in participants (e.g. smoking occurred only on the weekends, or only during the working week) suggesting that smoking in AHWs was not universally motivated by a biological addiction to nicotine. Nicotine dependence as a driver of smoking behaviours has been identified in community members and health care staff in a remote Indigenous community [32]. In this study we found a conscious awareness of ***Nicotine Addiction*** was present in only two interviewees. These individuals identified that they could sense when nicotine levels were low and that they smoked to “get the taste back” or “top up”.

### Family

The family is central to Aboriginal conceptualisations of health and wellbeing. Social and emotional wellbeing is defined as “a community where everyone feels good about the way they live and the way they feel” and dependent upon “connectedness to family, control over one’s environment and exercising power of choice” (p.6) [33]. Hence, when the health of an Aboriginal person’s family is threatened, so is their personal wellbeing. A range of features of family life increased the stress of AHWs and indirectly prompted smoking.

The burden of chronic conditions is great in Aboriginal communities [5], and stress due to ***Chronic Disease Burden*** in the families of AHWs was ever present and deeply felt in the stories shared. Respondents frequently spoke about the negative impact of heart disease, emphysema, diabetes, cancer and other diseases in their extended family.

The reduced life expectancy of the Aboriginal population [4] was reflected in comments made by participants. Many spoke of the death of family members due to chronic diseases and identified that distress due to ***Grief and Loss*** (particularly premature loss of parents) was associated with increasing the number of cigarettes smoked (in current smokers), or a relapse of smoking (in those that had quit).

Many AHWs also told of a high degree of ***Caring for Family*** responsibilities within their immediate and extended family. The three predominant responsibilities related to health support and advice, financial obligations and housing. Many AHWs spoke of the need to monitor the health of family members and provide management advice. Some AHWs shared that they were one of few individuals in their extended family who had a stable job and income, so their family frequently called upon them to loan money to those unemployed or on lesser incomes. The burden of providing extended family members with housing support was not commonly identified by AHWs, but for those that did have such responsibilities the impact on their wellbeing was dramatic.

"I used to have 18 people living with me and they were all on medication. I had a breakdown myself so I had to change my lifestyle."

Many AHWs spoke of happy marriages and family relationships and the support that their family provided in their lives. However, some AHWs identified that a ***Breakdown of Family Dynamics*** caused distress and prompted them to smoke. Isolation from family was one feature of disrupted family dynamics. It occurred in a geographical sense (as a result of moving away from home and family) and also occurred in the form of comprehensive alienation from family due to forced removal by government agencies. The lifelong trauma experienced by Aboriginal Australians forcibly taken from their family - known as Stolen Generations - has been well documented [34]. A relationship between forced removal and smoking has also been demonstrated elsewhere [35]. The second feature of disrupted family dynamics was divorced or single parent families. The supportive role of smoking in the life of a single parent is aptly described in this account:

"But yeah I’ve tried to give up smoking and because I don’t go out, I’m home all the time and I’ve got to have something to comfort me you know, because I’m a single mum trying to make it good for the children."

### Interpersonal processes

In interpersonal relationships, smoking emerged as a potent enabler of ***Socialisation and Connection***. Participants referred to smoking as a “social lubricant” that enhanced interactions with family, friends, co-workers, clients and strangers. In many health services, staff members commonly smoked and socialised together. In some settings terminology such as “the smokers” was used to describe those who congregated over a cigarette. Smoking has likewise been described as a “collective social practice” (p.60) [36] and “an activity linked to notions of belonging to a group” (p. 92) [37]. As one interviewee described in relation to AHWs:

"The smoking gives them a common bond with other people and it’s like you’re in a club."

Some participants felt that smoking was at times motivated by a need for a ***Debriefing Opportunity.*** That is, AHWs used smoking as a reason to leave the health service since it enabled a more private setting to talk through issues. The distinction between this and the ‘S*ocialisation and Connection’* motive is that in this instance smoking occurred following specific experiences that caused distress, whereas the socialisation motivation wasn’t related to a desire to talk about particular concerns. Individuals spoke of going outside to have a cigarette in order to “get stuff off their chest” or “let it out”.

Individuals are more likely to smoke if they have a twin, sibling, parent or friend who also smokes [38]. In our study, individuals expressed they smoked when they were exposed to ***Co-worker, Family, Friend or Client Encouragement.*** Many AHWs told of being surrounded by smokers in their family and social circles. Some noted that clients liked smoking with them, and that clients were more likely to be open and talk about their issues during a shared smoke. AHWs noted that at times people close to them would actively encourage them to go outside and smoke; however passive encouragement also existed where simply knowing that a person close to them was smoking prompted their desire to have a cigarette.

"You might be sitting down doing your work and then you get someone, “come out to have a smoke”, oh yeah, get up and go. So it’s just being around people, you know, being influenced by them I think."

Disputes and challenges in interpersonal communication emerged as a common stressor that promoted smoking behaviours in AHWs. First, there were some AHWs who observed their smoking was associated with ***Domestic Disputes.*** In one case, an AHW used smoking as a means to cope with the stress provoked by arguments and also as a reason to go outside to escape confrontation within the home. Second, a burdensome level of client expectation and in some cases unfair treatment by clients was described by a small number of respondents. Experiences of ***Client Expectations and Ill-treatment*** were described by AHWs where clients refused to be treated by them, accused them of breaching confidentiality or expected them to “always find the solution”. Third, a negative effect of a top-down approach to policy implementation was evident. Specifically, ***Lack of Communication between Policy Makers, Management and Health Workers*** was identified as a source of stress for some AHWs since they were left with an appreciation of *what* had to be done but not *why.* Finally, a majority of AHWs and other health service employees spoke of supportive and collaborative relationships with colleagues. In some instances, though, disagreements with co-workers or lack of acknowledgement by co-workers were identified as stressors that promoted smoking, as was being micromanaged by a more senior colleague. ***Staff Disputes and Micromanagement*** were more commonly mentioned by respondents working in government-controlled health services, possibly reflecting greater intercultural challenges between AHWs and their non-Aboriginal colleagues in these settings.

"I was micro managed at work and after so long of being micro managed and not getting supported, I actually increased dramatically the smoking and it wasn’t good."

### The health service

There were a range of features of work and the work environment that increased the stress levels of AHWs. In times of heightened stress, some AHWs reported using smoking as a means to cope. Elaboration of work-related stressors is therefore necessary to understand the full range of factors that indirectly perpetuate smoking in AHWs. ***Demanding Work*** was frequently reported as a source of stress that triggered smoking. Features of demanding work included: excessive scope of practice and lack of role clarification; complex practice (e.g. dealing with multiple health issues); insufficient resources; excessive workload; difficulties in having positive impact on health outcomes; and challenges in bridging the cultural gap. Work demands varied greatly, however, and were dependent upon the work setting, the job specifications of the AHW, and their experience. The demands of an excessive scope of practice, commonly mentioned by respondents, is mirrored by the reflections of Mitchell and Hussey [8] who describe that AHWs are "asked to take on many roles at once” and be “everything to everyone” (p.529). Many AHWs also described burdensome ***Out of Hours Work*** when community members approached them for advice in public spaces, such as the supermarket, or even came to their home. This latter occurrence was mentioned by AHWs working in small communities.

"I was getting a few of my clients starting to come home and wanting to get support and even though I did that, that would impact on me at times … and then I went back smoking again."

A predominance of short-term contracts and uncertainty regarding contract renewals lead to some concerns about ***Job Insecurity and Financial Insecurity***. Precariousness of employment caused financial stress in AHWs due to concerns about meeting financial obligations and obtaining bank loans. Participants from two different regional areas identified ***Salary Disparity*** as another source of stress. Specifically, inequity in pay between AHWs and other health staff (such as registered nurses) or between AHWs in community-controlled versus government-controlled health services was distressing. Similarly, Mitchell and Hussey [8] observe that “remuneration in the community sector is not progressing in line with government-employed health workers” (p.530). In some health services, general ***Workplace Instability*** was troubling. The predominant features of workplace instability included frequent changes in management and loss of staff. Such cut backs were described by respondents employed in two health services visited in this study.

"Yeah look we haven’t had a solid foundation in the last 2 years, it’s changing, this is worse than ‘Days of our Lives’ [soap opera] coming into this place, something’s different everyday, you know."

***No Training or Promotion or Staff Support*** was identified as a stressor by some AHWs who were concerned by a lack of opportunity to progress in their career. Inequity in employment and training opportunities for the Aboriginal compared with non-Aboriginal health workforce has previously been identified [39].

"I’ve seen the effect it has on new Aboriginal health workers that feel a sense of powerlessness, a sense of hopelessness within their own workplaces so our feeling is that we are just forced to be kept at a certain level and we’re not allowed to go beyond a certain line."

Stress due to ***Institutionalised racism and cultural insensitivity*** was acutely felt by some AHWs. In one instance an AHW described being judged and mistreated by employees of social welfare services. Racism was commonly experienced in government-controlled health services through the comments and actions of non-Aboriginal staff.

"It’s probably in a work context as well, massive institutionalised racism, I’ve not just seen it here; I’ve seen it in a lot of places I’ve worked. I’ve even had it directed at me, comments like are you 5% Aboriginal?"

### The community

At the community level, a range of stressors were reported by AHWs and other participants. ***Dispossession of land*** was reported by a small number of respondents as an injustice that continues to disadvantage Aboriginal families. AHWs spoke of the negative implications of lack of land ownership for future generations who have no inheritance (such as reduced financial opportunities and education). Also, while grief and loss was articulated at the family level, ***Collective Grief and Loss*** had negative repercussions at the community level.

"There’s a lot of grief and loss and talk about collective trauma that’s in Indigenous communities and populations and people come in and one of the ways they'd, you know, debrief was to sit down have a yarn and, you know, have a cigarette perhaps while they’re doing it."

Prevalent ***Racism*** in the community was described by some AHWs in the forms of name calling, denial of cultural identity and discrimination (in relation to housing, job seeking and when attempting to use community services). Social marginalisation and racism is believed to lead to self-harming and substance misuse [40]. In fact, systematic review evidence demonstrates that self-reported racism is consistently associated with smoking tobacco [41].

Participants were commonly distressed by the array of ***Social Disadvantage*** characteristics present in Aboriginal communities. Features such as poverty and homelessness, unemployment, chronic disease, drug and alcohol abuse, housing issues, gambling, violence, imprisonment and lack of education and recreation opportunities were identified both in the general community and in family members (highlighting the proximity of social disadvantage to AHWs). Empirical data demonstrates an association between social economic position and smoking in Indigenous Australians. Higher social economic position, assessed using nine separate markers (such as income, education, employment, financial stress, etc.) is consistently related to non-smoker status [35].

### Public policy

***Short Term and Insecure Funding*** was a macro level stressor infrequently described by respondents. Funding insecurity perpetuates other proximal stressors such as job insecurity and workplace instability that promote smoking behaviours through creating stress in AHWs. The challenges of funding insecurity were described in this account:

"If you want to build an economic stability for yourself, don’t build it around an Aboriginal organisation, because we’re fearful that if our board stuffs up, they’ll take the money away from us. If we stuff up, they’ll take the money away from us. If our community disagrees with the way that we’re going, they’ll take the money away from us. [Note: the term “stuff up” is an Australian colloquialism referring to inadequate practices such as mismanagement or clinical error]"

### Thematic summary

Following collective consideration of the data, five underlying themes emerged. First, experiences of ***Stress*** predominated in the stories about smoking. Stressors were perceived in multiple contexts and were an ever-present feature of life. There was a sense of ***Burden*** in the lives of AHWs due to the multitude of stressors faced and the extensive responsibilities and obligations within the family, the workplace and the community. In deconstructing and examining the events and experiences that precipitated stress it is evident that many were outside the control of AHWs. For example, AHWs spoke of an inability to exert influence over the premature death of loved ones, job insecurity, inadequate training and promotion opportunities and institutionalised racism. Hence a sense of ***Powerlessness*** prevailed in the stories shared by AHWs. A perceived ***Lack of Value and Recognition*** was also observed where AHWs spoke of getting the blame if anything went wrong and not being acknowledged for what they do. When considered together, the experiences of client mistreatment, lack of training or promotion opportunities, micromanagement, inequitable salaries and job insecurity demonstrated to AHWs that they were not valued within the health care system. Finally, ***Connectedness,*** or the lack thereof, influenced smoking behaviours in a range of environments. Specifically, smoking was motivated by a desire for connectedness (e.g. socialising, debriefing) and also prompted by distress due to a lack of connectedness (e.g. breakdown of family dynamics, staff disputes). In summary, smoking fulfilled two primary functions in AHWs including providing a means to enable socialisation and connection and a means to cope with stress, burden, powerlessness, lack of value and recognition, and disconnection. Smoking therefore is perpetuated by the accepted use of cigarettes to promote socialisation and connection in Aboriginal society and the multitude of personal, social and environmental stressors faced by AHWs.

## Discussion

The stories shared revealed an expansive and complex spectrum of factors that were associated with smoking in AHWs. A majority of AHWs spoke of a desire to quit smoking, but told of many factors that prompted and reinforced smoking in their lives. Nicotine addiction and individual associative behaviours are but one small part of the story. Rather, multilevel and interrelated sources of stress were evidenced by countless accounts of the burden of chronic disease, family obligations, workplace instability, job demands, social disadvantage and racism, to name but a few. In this context, smoking was used by AHWs as a means to cope with the multitude of life’s challenges. These findings highlight that to view smoking through the lens of nicotine addition and individual choice is myopic. Consistent with prevailing approaches to promoting health in Indigenous populations, a holistic social ecological paradigm clearly positioning the individual within their environmental context is essential to understand the factors that perpetuate smoking in AHWs [22,42].

AHWs were cognisant of being members of a small and disadvantaged population, and described experiences of racism and discrimination. Institutionalised racism and micromanagement were stressors more commonly identified by AHWs working in government-controlled health services compared to community-controlled organisations. The predominance of these stressors in government health services - where the Aboriginal health team sits within a larger non-Aboriginal workforce - may be precipitated by the tarnished history of colonisation that perpetuates a lack of trust between Aboriginal and non-Aboriginal Australians. It could be that the chronology of repeated abuse, disrespect and dispossession since white settlement and the ongoing traumatisation due to racism and disadvantage may propel Aboriginal people towards seeking connection and belonging through smoking.

A majority of AHWs spoke of widespread acceptance of smoking in Aboriginal families and in the community. The extensive history of nicotine ingestion in traditional Aboriginal society coupled with the promotion of tobacco habituation during colonisation has likely contributed to a normalisation of smoking in contemporary Aboriginal society. Daily exposure to smoking at home, at work or in the community provided constant reminders and frequent opportunities for AHWs to smoke with others. The key role of smoking in promoting social connectivity found in AHWs is consistent with the view of Roche and Ober [43]: ‘Sharing a cigarette has also become one of the ways in which indigenous people have been able to reaffirm, strengthen and maintain their cultural identity in an environment that is often hostile and constantly changing’ (p. 130). Though smoking was viewed as negative in terms of the long-term health impacts, in the short-term it was viewed by some AHWs as an effective health promoting behaviour. There were some respondents who felt that smoking was the only thing in their life that made them feel calm, enabled them to connect with others, and helped them cope with the stress and responsibilities in their life. Similarly, smokers in the general population report benefits from smoking (such as socialisation, stress management and relief from boredom) [37]. Clearly, any strategies to support AHWs to quit smoking must provide healthful alternative means to promote social cohesion and manage stress.

Knowledge of the multilevel factors associated with smoking in AHWs can inform the development of health service smoking cessation programs. Our data suggests that multidimensional programs are needed that target individual factors as well as the social and environmental factors perpetuating smoking in AHWs. This is in line with ecological approaches to prevention aimed at reducing the burden of cardiometabolic diseases in Aboriginal populations by addressing the direct and indirect factors that perpetuate behaviours like smoking [42]. To address the direct response that comes from the habituation of smoking, the findings provide key biological and behavioural targets at the individual level (e.g. *Nicotine AddictionAssociative BehavioursStress*) that may be ameliorated through such strategies as nicotine replacement therapy and counselling (e.g. counselling regarding behaviour change strategies and stress management). A number of strategies can be mounted to address the indirect environmental factors associated with smoking. At the interpersonal level, *Co-worker, Family, Friend or Client Encouragement* could be addressed through culturally-relevant social marketing campaigns and smokefree workplace policies that challenge the normalisation of smoking at home and at work. Organisational stressors such as *Demanding Work* and *Institutionalised Racism and Cultural Insensitivity* could be addressed through organisational change processes where the health service clarifies the scope of practice of AHWs and institutes cultural competency training for non-Aboriginal staff to promote cultural respect in the workplace. In future work, researchers will engage stakeholders to identify culturally-relevant strategies that address the multilevel factors that perpetuate smoking in order to promote cessation and abstinence in AHWs.

The factors associated with smoking for a given individual were uniquely distributed, depending upon their personal characteristics, relationships and environmental context. Hence, programs that can be tailored to local needs are indicated. Given that we comprehensively collected the perspectives of AHWs from a broad range of settings within government- and community-controlled health services, the findings of the study are potentially transferable to AHWs and health professionals in a similar role in other regions across Australia. The findings may also be relevant to Indigenous populations in other countries sharing a similar history of colonization and tobacco use.

Despite a range of strengths, this work has limitations to consider. The interviewers were of non-Aboriginal descent, and this cultural difference could have negatively impacted the data collection process. Perhaps because the Tackling Tobacco Coordinator vouched for the sincerity and credibility of the research team, and because there was a respected Aboriginal person as a chief investigator on the project, the interviewees appeared comfortable with interviewers and were happy to share intimate details of their experiences. The trust afforded by participants may reflect the strengths of the participatory process that guided the project methodology, such as taking the time to develop relationships with the health services and returning the findings to AHWs and Aboriginal stakeholders for interpretation and refinement [18]. However, there are likely to be some individuals who chose not to participate in interviews and discussions because they did not feel comfortable talking about their personal life with a non-Aboriginal researcher. Further, we are likely to have missed the personal views of individuals who felt sensitive about their smoking addiction and unwilling to discuss it (as they would not have volunteered to interviews). The use of shadowed data in this work [25], where respondents shared stories about the experiences of others, compensates in part for this. In one example, an AHW ex-smoker shared her observations of the quitting challenges of an AHW colleague (who hadn’t volunteered to participate) who was stressed by the workplace environment.

## Conclusions

In summary, an extensive array of factors directly and indirectly perpetuated smoking in AHWs. The multitude of personal, social and environmental stressors faced by AHWs and the accepted use of communal smoking to facilitate socialisation and connection were primary drivers of smoking in AHWs in addition to nicotine dependence. Culturally sensitive multidimensional smoking cessation programs that address these factors are needed. The factors associated with smoking were uniquely distributed for individual AHWs depending upon their personal characteristics, relationships and environmental context. Therefore, programs that can be tailored to local needs are indicated.

## Competing interests

The authors declare that they have no competing interests.

## Authors' contributions

AD undertook data collection and analysis and drafted the manuscript. HS provided cultural mentoring during the study, assisted in analysis and refined the manuscript. AC participated in the design and coordination of the study, assisted in analysis and refined the manuscript. MC and MD conceived of the study, were responsible for its design and coordination, and refined the manuscript. MC also undertook data collection and secondary review of the analysis. All authors read and approved the final manuscript.

## Pre-publication history

The pre-publication history for this paper can be accessed here:

http://www.biomedcentral.com/1472-6963/12/102/prepub
